# Prospective enterovirus D68 (EV-D68) surveillance from September 2015 to November 2018 indicates a current wave of activity in Wales

**DOI:** 10.2807/1560-7917.ES.2018.23.46.1800578

**Published:** 2018-11-15

**Authors:** Simon Cottrell, Catherine Moore, Malorie Perry, Ember Hilvers, Chris Williams, Ananda Giri Shankar

**Affiliations:** 1 Public Health Wales Health Protection Division, Number 2 Capital Quarter, Tyndall Way, Cardiff, Wales, United Kingdom; 2 These authors contributed to the work equally and share first authorship; 3 Public Health Wales Microbiology Division, University Hospital of Wales, Heath Park, Cardiff, Wales, United Kingdom

**Keywords:** enterovirus, D68, respiratory, surveillance, acute flaccid myelitis

## Abstract

Since 7 June 2018, an enterovirus D-68 (EV-D68) season (the third since 2015) is ongoing in Wales, with 114 confirmed cases thus far. Median age of the 220 cases since 2015 is 2.5 years (2.5 years in intensive care cases), 94% were hospitalised, 17% (n = 38) in intensive care. All had respiratory symptoms; bronchiolitis symptoms were reported in 60 cases, severe respiratory symptoms in 23 and acute flaccid myelitis in two cases.

A seasonal wave of enterovirus D68 (EV-D68) infection is ongoing in Wales, the first case was detected on 7 June 2018. This is the third EV-D68 season detected in Wales since 2015. Here, we present data on the current and two previous EV-D68 seasons. Recent surveillance data prompted a pro-active EV-D68 electronic alert sent to paediatricians, other clinicians and microbiologists in Wales.

## Enterovirus D-68 surveillance and case definition

In 2015, a cluster of 10 adult and four child (< 16 years) acute flaccid myelitis (AFM) cases was detected in Wales [1], EV-D68 (n = 2) and Echovirus 25 (n = 1) were identified from three child cases. This led to implementation of enhanced surveillance for enterovirus-associated AFM, prospective laboratory-surveillance of enterovirus in respiratory specimens and genetic characterisation of all laboratory-detected enteroviruses in Wales.

Prospective laboratory-based passive surveillance for EV-D68 is carried out as part of wider enterovirus surveillance. All samples submitted from patients with respiratory symptoms, including those from the Welsh sentinel General Practitioner (GP) surveillance network (which covers around 400,000 (13%) people in Wales) are tested as part of a wider respiratory panel test using the Luminex NexTag Respiratory Pathogen Panel (RPP) molecular assay and a specific assay targeting the 5’-non-coding region (NCR) of enteroviruses with internal control. This allows differentiation of rhinovirus and enterovirus. Enterovirus-positive samples (including dual-positive samples) are then tested by in-house EV-D68 specific real-time polymerase chain reaction (PCR), based on described methods [2]. Enterovirus-positive stool samples were typed using partial VP1 sequencing [3].

In all, 1,026 patients had confirmed enterovirus infections from 1 September 2015 to 5 November 2018, including those confirmed from non-respiratory samples. Enterovirus typing was possible in 861 (83.9%) of samples, of which EV-D68 was the most commonly detected type (25.6%). Other common types included: Coxsackie virus A6 (16.0%), Echovirus 30 (14.2%) and Echovirus 6 (9.1%); A71 accounted for 2.3% of typed enteroviruses.

Five cases of AFM were detected with enterovirus infection during this period, two with EV-D68, two with A-71 and one with Echovirus 25. AFM was ascertained based on acute onset of flaccid limb weakness, with neurological imaging suggestive of myelitis.

We defined an EV-D68 case as any patient with respiratory symptoms and a respiratory, cerebrospinal fluid (CSF) or stool sample with PCR/sequencing evidence of EV-D68.

For each case, available clinical features (at sample collection) were ascertained from free-text on test request forms and recorded in a national database, along with location (community or hospital), standard demographic details and underlying risk factors if available. Completeness of data presented here is likely to be lower for weeks 41 to 44, the proportion of symptoms reported and ICU confirmations during season three may increase as late data are received.

## Enterovirus D-68 surveillance findings

From 1 September 2015 to 5 November 2018, there were 220 cases of EV-D68 infection confirmed in Wales (average incidence of the three seasons was 2.3/100,000 population) of which, 212 were from respiratory tract samples (predominantly throat or nose swabs and fluid), two were from faecal samples and sample type was not available for six. Of the cases, 93.6% (206/220) were hospital patients, including 38 (17.3%) in intensive care units (ICU). The remaining 14 samples were submitted by sentinel GPs (n =2), non-sentinel GPs (n = 2), pathologists (n = 2) or unknown settings (n = 8). Most cases were hospitalised with respiratory symptoms.

## Seasonality

Cases occurred in three distinct seasons (Figure, Table 1). In the first (11 December–25 February) there were 56 cases (1.8/100,000 population), with a peak in late January. In the second (6 July 2016 to 3 January 2017) there were 48 cases (1.5/ 100,000 population) with a peak in late July. Very little EV-D68 activity was seen during 2017, with only two sporadic cases occurring in October. In the third (current) season, still ongoing as at 5 November, the first case was detected on 7 June 2018 and 114 cases have been confirmed thus far (3.6/100,000 population). The peak in weekly numbers of confirmed cases was during October 2018.

**Figure:**
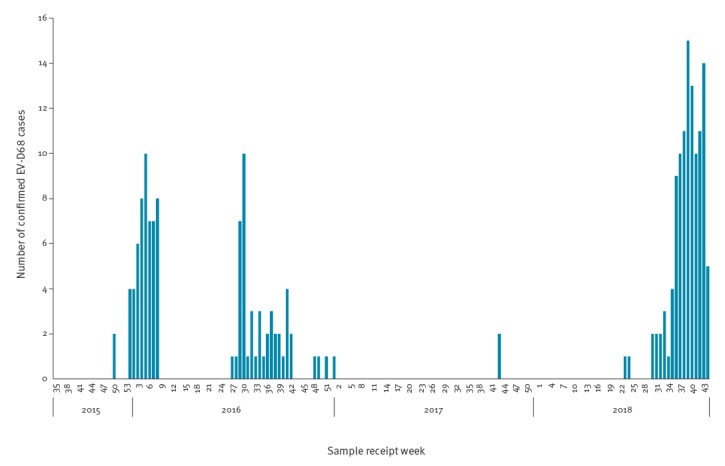
Confirmed enterovirus D-68 cases, Wales, week 35 2015–week 44 2018 (n = 220)

**Table 1:** Characteristics of seasons of enterovirus D-68 activity, Wales, 1 September 2015–5 October 2018^a^

	Season one (11 Dec 2015–25 Feb 2016)	Season two (6 Jul 2016–3 Jan 2017)	Season three (7 Jun 2018–present)^a^	Total^b^
**Total number of cases**	**56**	**48**	**114**	**220**
Duration (weeks)	10.9	25.9	16.3	Ongoing
Female (n)	35	26	44	106
Mean age in years (range)	11.3 (0.1–71.8)	16.0 (0.0–85.6)	20.0 (0.0–101.0)	16.9 (0.0–101.0)
Median age in years	1.7	3.1	3.5	2.5
**Samples from ICU**				
Female (n)	7	6	10	24
Mean age in years (range)	11.1 (0.1 - 63.0)	30.5 (0.6 - 82.2)	32.4 (0.0 - 81.2)	25.8 (0.0–82.2)
Median age in years	0.9	8.1	16.5	2.5
**Total ICU cases**	**12**	**7**	**18**	**38**

^a^ Ongoing as at 5 November 2018.

^b^ Total includes two sporadic detections during 2017.

Although regional differences in testing patterns may influence comparisons of case numbers between areas, cases were seen in all regions of Wales in each enterovirus D-68 season.

Of 220 cases, 61% were children younger than 5 years and the median age was 2.5 years. However, cases occurred in all age groups and the range of ages affected has widened with each season. During the second and third season, ICU cases tended to be older. Samples from ICU accounted for 38/220 cases (Table 1).

The relative risk (RR) of being confirmed in ICU was 2.56 (95% CI: 1.08–6.13, p value: 0.03) for patients aged 65 years and older, compared with adults aged 18–64 years (Table 2). Overall cases were equally distributed between males and females (114 and 106% respectively), however females accounted for more ICU cases than males (24 and 14 respectively).

**Table 2:** Confirmed cases of enterovirus D-68 by age group, Wales, September 2015–October 2018^a^ (n = 220)

Age (years)	All cases		Confirmed in ICU	
	Total	Average seasonal incidence per 100,000 population	Total	RR (95% CI)
Children < 5	136	26.4	21	1.03 (0.47–2.28)
Children 5–17	16	1.2	2	0.84 (0.19–3.63)
Adults 18–64	47	0.8	7	Ref
Adults 65 and older	21	1.1	8	**2.56 (1.08–6.13)**
**Total**	**220**	**2.3**	**38**	**NA**

CI: confidence interval; ICU: Intensive care unit; NA: not applicable; RR: relative risk.

^a^ Number confirmed in ICU and relative risks for being an ICU case compared with adults aged 18–64 years: RR with significant p values are shown in bold.

Although regional differences in testing patterns may influence comparisons of case numbers between areas, cases were seen in all region of Wales in each EV-D68 season.

## Clinical Setting and Symptoms

In addition to general respiratory symptoms, other reported symptoms included fever (65% of cases, 20/38 of ICU cases), wheeze/bronchiolitis (27% of cases, 6/38 of ICU cases), pneumonia/requirement for ventilator support (10% of cases, 19/38 of ICU cases) (Table 3). Other severe symptoms included sepsis or organ failure (5% of cases, 3/38 of ICU cases) and AFM/AFP (1% of cases, 1/38 of ICU cases).

**Table 3:** Reported symptoms by age group and intensive care unit status (at time of sampling) in confirmed cases, Wales, 1 September 2015–5 November 2018^a,b^ (n = 220)

Reported symptom	Age group (years)	Confirmed cases with symptom (n = 220)		ICU cases with symptom (n = 38)
		n	%	n
Wheeze/ crackles or bronchiolitis	Children < 5	57	41.9	6
	Children 5–17	2	12.5	0
	Adults 18–64	0	0.0	0
	Adults 65 and older	1	4.8	0
	**Total**	**60**	**27.3**	**6**
Fever	Children < 5	84	61.8	11
	Children 5–17	12	75.0	2
	Adults 18–64	37	78.7	5
	Adults 65 and older	9	42.9	2
	**Total**	**142**	**64.5**	**20**
Rash, vesicles or hand foot and mouth symptoms	Children < 5	2	1.5	0
	Children 5–17	0	0.0	0
	Adults 18–64	0	0.0	0
	Adults 65 and older	0	0.0	0
	**Total**	**2**	**0.9**	**0**
Pneumonia, requiring ventilation or with respiratory failure	Children < 5	9	6.6	9
	Children 5–17	2	12.5	2
	Adults 18–64	6	12.8	3
	Adults 65 and older	6	28.6	5
	**Total**	**23**	**10.5**	**19**
Sepsis or organ failure	Children < 5	7	5.1	1
	Children 5–17	0	0.0	0
	Adults 18–64	3	6.4	1
	Adults 65 and older	2	9.5	1
	**Total**	**12**	**5.5**	**3**
Acute Flaccid Myelitis	Children < 5	2	1.5	1
	Children 5–17	0	0.0	0
	Adults 18–64	0	0.0	0
	Adults 65 and older	0	0.0	0
	**Total**	**2**	**0.9**	**1**

ICU: intensive care unit.

^a^ See Table 2 for age group denominators.

^b ^Symptom data is reported as at 7 November 2018 and may under-report for weeks 41–44.

## Discussion and implications

The surveillance data here provide evidence of current EV-D68 circulation in Wales. EV-D68 may have also circulated in other countries of Europe in recent months, however lack of evidence from surveillance schemes makes it difficult to judge how widespread circulation is. Increasing awareness and better diagnostic and virus characterisation techniques have improved ascertainment of EV-D68 over the past decade. However, despite there being many published outbreak reports on EV-D68, there remains a paucity of population-based surveillance that could be used to describe the disease burden, range of syndromes caused, risks of acquisition and of severe outcomes [4]. Arrangements for routine enterovirus diagnostic typing are also highly variable [5,6]. These factors lead to underestimation of the burden and circulation of enterovirus and biased information towards cases from retrospective outbreak investigations.

The prospective surveillance data here indicate that there have been three widespread seasons of EV-D68 in Wales since 2015, lasting from 10 to 25 weeks. The temporal distribution of EV-D68 activity in Wales is broadly similar to reports from outbreaks previously described elsewhere, including emerging reports of EV-D68 activity and AFM in 2018 from the US [7,8]. The first season was unusual compared with subsequent ones in its timing and may have had a lower age distribution, the reasons for this are unclear but could be consistent with the arrival of emerging EV-D68 within Wales.

A study of 30 severe enterovirus infections in children from 2012–13, in the south of England did not find any association with EV-D68 [9]. A retrospective study reporting typing results for 2,770 enterovirus samples submitted to the Public Health England Virus Reference Department during 2004–11, indicated that EV-D68 was only detected in 14 of 1,875 typed samples [10]. This suggests that although EV-D68 has been historically seen in the UK, there has been a comparative increase since 2014. Population incidence of confirmed cases in Wales is highest for children younger than five years, as is the number of ICU cases; however the risk for ICU admission appears higher in adults aged 65 years and older. Respiratory symptoms were widespread, but in line with previous reports [11-14], severe outcomes were most frequent in the very young and in older adults.

The data here are likely to under-represent the true circulation of EV-D68 and other enteroviruses in Wales, as most patients with acute respiratory symptoms, will not undergo testing. In addition, it is likely that EV-D68 in the community was mostly undetected, due to potential under-ascertainment of mild cases and under-sampling. Only two cases, both presenting with influenza-like illness, were from the Welsh sentinel GP virological surveillance network. Most EV-D68 cases were identified from hospitals, which may have overestimated the severity of infection, with only severe cases being hospitalised, and also biased findings towards child cases. The age-profile of EV-D68 hospitalised cases is potentially due to young children and older adults being most at risk of severe acute respiratory infections.

Clinical information is not consistently provided with samples. Therefore, improving and standardising the recording of patient risk factors and clinical symptoms in sample test-request forms would provide useful information on the burden, risks and acquisition of EV-D68. Advising clinicians on the respiratory presentations of EV-D68 and alerting them to known active circulation will help improve current under-ascertainment and consistency of recording. In addition, enhanced surveillances and/or follow-up studies on EV-D68 cases are needed to better understand the risks, duration of illness, length of hospital stay, patient outcomes and long-term sequelae.

The prevalence of severe complications and paucity of systematic surveillance data reinforce the importance of continuing and improving surveillance in this area and implementing recent enterovirus diagnostic guidance for systematic surveillance of non-polio enterovirus types [5]. While there are limitations with the current EV-D68 surveillance activities in Wales, there has been consistency in the testing approach and the inclusivity of all patients tested for acute respiratory infection. International collaboration is needed for a better understanding of the temporal and geographical pattern of circulation.
